# ATP modulation of osmotically activated anionic current in the membrane of *Phycomyces blakesleeanus* sporangiophore

**DOI:** 10.1038/s41598-023-39021-9

**Published:** 2023-07-24

**Authors:** Katarina S. Stevanović, Bogdana Čepkenović, Strahinja Križak, Tanja Pajić, Nataša V. Todorović, Miroslav Ž. Živić

**Affiliations:** 1 Faculty of Biology, Institute of Physiology and Biochemistry, University of Belgrade, Studentski Trg 16, Belgrade, 11158 Serbia; 2 Institute of Multidisciplinary Research, University of Belgrade, Kneza Višeslava 1, Belgrade, 11030 Serbia; 3 Institute for Biological Research “Siniša Stanković”, University of Belgrade, National Institute of the Republic of Serbia, Bulevar Despota Stefana 142, Belgrade, 11000 Serbia

**Keywords:** Biophysics, Cell biology, Microbiology, Physiology

## Abstract

Ion channels are vital components of filamentous fungi signaling in communication with their environment. We exploited the ability of the apical region of growing sporangiophores of *Phycomyces blakesleeanus* to form membrane-enveloped cytoplasmic droplets (CDs), to examine ion currents in the filamentous fungi native plasma membrane. In hypoosmotic conditions, the dominant current in the CDs is ORIC, an osmotically activated, anionic, outwardly rectified, fast inactivating instantaneous current that we have previously characterized. Here, we examined the effect of ATP on ORIC. We show that CDs contain active mitochondria, and that respiration inhibition by azide accelerates ORIC inactivation. ATP, added intracellularly, reduced ORIC run-down and shifted the voltage dependence of inactivation toward depolarized potentials, in a manner that did not require hydrolysis. Notably, ATP led to slowing down of ORIC inactivation, as evidenced by an increased time constant of inactivation, τ_in_, and slower decline of τ_in_ during prolonged recordings. Flavonoids (genistein and quercetin) had the effect on ORIC opposite to ATP, acting as current inhibitors, possibly by disrupting the stabilizing effect of ATP on ORIC. The integration of osmotic sensing with ATP dependence of the anionic current, typical of vertebrate cells, is described here for the first time in filamentous fungi.

## Introduction

Filamentous fungi, ubiquitous organisms in the environment, can respond and adapt to a wide range of varying surrounding conditions. Yet, very little is known about the physiological mechanisms mediating their adaptability at the level of the cell membrane. Scarcity of studies addressing fungal membrane physiology is likely a direct consequence of experimental difficulties for electrophysiological measurements of ion currents^1^, rather than a lack of interest, since ecological^2–4^, biotechnological^5,6^ and biomedical^7–10^ significance of fungal physiology is undisputed.

Cytoplasmic droplets obtained from *Phycomyces blakesleeanus* sporangiophores have been used as an experimental model by our research group, for high-quality patch clamp measurements on a native fungal plasma membrane^1,11^. *P. blakesleeanus* is a saprophytic filamentous fungus from the order Mucorales, and can be considered as a proxy model system for the basic physiology of its medically and economically important cousins from the order Mucorales^12–14^.

The sporangiophore *of P. blakesleeanus* is a giant unicellular multinucleated aerial hypha, well known for classical research on its light and gravity response coupled with growth^15,16^. Cytoplasmic droplets (CDs) are obtained from *P. blakesleeanus* sporangiophore, through spontaneous formation upon releasing the content from the tip of the sporangiophore in an adequate solution, as first described by Zaichkin et al.^17^. Cytoplasmic droplets obtained this way are membrane-bound and have the potential to regenerate the cell wall^1,17^.

An outwardly rectifying, instantaneous, fast-inactivating current (ORIC) is a dominant current in the cytoplasmic droplet membrane under hypoosmotic conditions^1^. It shares some of the key features that define the biophysical signature of the vertebrate volume-regulated anion channel (VRAC): osmotic activation, mild outward rectification, selectivity for anions following Eisenman I series and inactivation at depolarizing potentials that resemble many of the VRAC isoforms^18^. One of the most striking features of ORIC is the fast rundown accompanied with an increase in the speed of its inactivation^1^. Notably, all of these properties were discerned under the conditions of dialysis of the cytoplasmic droplet by patch pipette solution that lacked ATP.

We hypothesized that inclusion of ATP to the pipette solution may be necessary to better mimic the physiological environment. In addition, ORIC was shown to be sensitive to anthracene and niflumic acid^1,19^, anionic channel blockers that also inhibit respiration and growth of *P. blakesleeanus* sporangiophore^19^, suggesting a possible link between metabolism and ORIC. ATP, beside acting primarily as a fuel for kinases that either directly phosphorylate the ion channel or trigger a signaling cascade that leads to channel modulation, is also known to act as an allosteric modulator of many proteins^20^. So far, several anion channels, including VRAC, are shown to be regulated by direct binding of ATP^21,22^. Here, we have investigated the modulation of ORIC properties by ATP and other molecules known to modify ATP availability or interact with ATP-binding sites. The data obtained suggest that ORIC, like VRAC in vertebrates, is regulated by ATP and that the presence of ATP is necessary for sustained ORIC activity.

## Results

### ORIC run-down can be reduced or completely abrogated by addition of ATP to pipette solution

**Figure 1:**
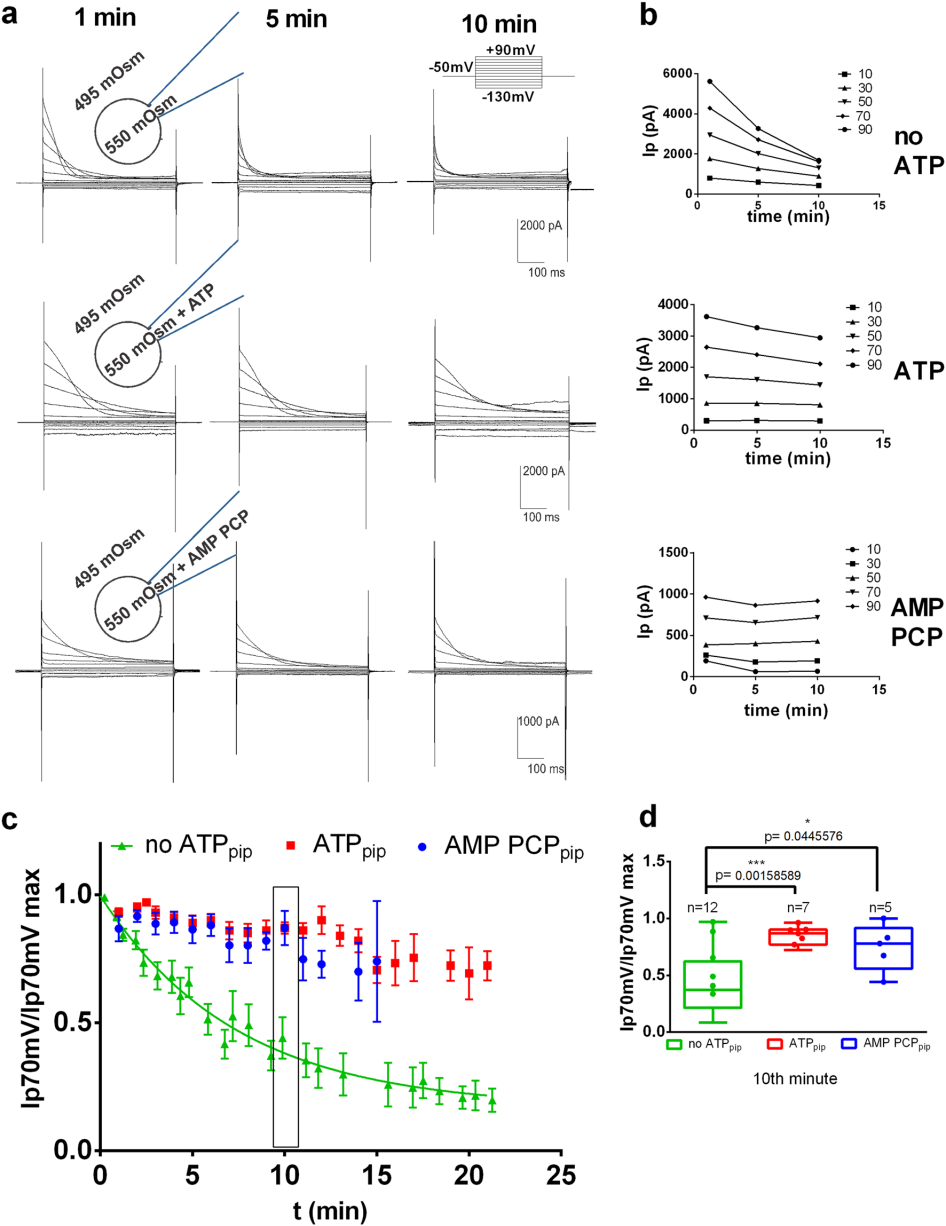
ORIC run-down is dramatically reduced by ATP_pip_ and the non-hydrolysable ATP analogue AMP PCP_pip_. (**a**) Representative recordings of ORIC voltage family at 2, 5 and 10 min after whole cell break in: top row—control conditions (without ATP added); middle row—2 mM ATP in pipette; bottom row—2 mM AMP PCP in pipette. Ip and Iss were measured at the beginning and at the end of the response to voltage pulse, respectively, avoiding capacitive transients. Voltage stimulation waveform for standard voltage protocol is depicted in top right corner. Schematic of experiment, with osmotic conditions and compounds in the pipette, present throughout the time of the recording, is shown on the far left side in each row. (**b**) Plots generated from representative recordings shown in a. Time dependence of peak current values at depolarizing potentials (+10 to +90 mV) emphasizing that ORIC run-down in the absence of ATP is present at all potentials that are inducing current inactivation, while it is mitigated by ATP and AMP PCP. (**c**) Time dependence of peak current at + 70 mV (Ip70 mV), normalized to maximal value obtained for that individual recording series. Mean ± SE, with exponential fit of no ATP_pip_ series (half-life 95% confidence interval: 3.6–8 min). Extra-sum-of-squares F-test of time dependence of all three series, p < 0.0001. n = 32 (no ATP_pip_), 25 (ATP_pip_), 11 (AMP PCP_pip_). Rectangle marks the time point analyzed in (**d**). (**d**) Box and whisker plots enclosed by the 25th and 75th percentile range, median line with whiskers extending from minimal to maximal value, all points shown, of normalized peak current at + 70 mV, at 10 min. The number of points and probabilities from comparison to no ATP_pip_ group is shown. ANOVA following the Holm–Sidac correction for multiple comparisons.

Osmotically-induced anion currents corresponding to ORIC were elicited by 500 ms voltage steps from a holding potential of − 50 mV. As described previously^1^, inactivation of the outward Cl^-^ currents in the absence of ATP in pipette solution is evident starting from the + 10 mV pulses (Fig. 1a, top row), and the decrease in current amplitude with repeated depolarization stimuli is evident at all potentials (Fig. 1a,b, top row). Normalized amplitude of the peak current at +70 mV is characterized with exponential run–down, reaching approximately 25% of the initial value 20 min after whole-cell breaking through the membrane (Fig. 1c, green symbols). Addition of 2 mM ATP to the pipette solution (ATP_pip_) stabilized ORIC amplitude, resulting in statistically significant decrease of the current run-down (Fig. 1a,c, red symbols, p < 0.001). Addition of 2 mM Mg^2+^ in pipette solution without ATP had no effect on ORIC, so data were pooled with (no Mg^2+^) no ATP group. Replacement of ATP with the nonhydrolyzable ATP analogue AMP PCP (AMP PCP_pip_) had the same stabilizing effect on ORIC, indicating that ATP binding is sufficient for the effect, whereas ATP hydrolysis is not required (Fig. 1a,c, blue symbols). When we compared the normalized values of peak currents at + 70 mV at the 10 min time point (Fig. 1d), we found that both ATP and AMP PCP maintained the ORIC amplitude above 70% of the initial value (mean ± SD values: 85 ± 8% for ATP and 75 ± 20% for AMP PCP), while in no ATP group it declined (mean ± SD: 44 ± 28%).

We also tested if 2 mM GTP affects ORIC in a similar manner as ATP (as shown in Supplementary Fig. 1a). Normalized GTP_pip_ current time-course (n = 6) overlaps with the curve for the current without ATP and has significantly different speed of exponential decay (p = 0.0182) than with ATP_pip._ After 6 and to 7 min, GTP_pip_ ORIC current is reduced to 62 ± 17%, significantly larger reduction compared to ATP_pip_ (p = 0.014) (Supplementary Fig. 1b), demonstrating that GTP can not substitute ATP as a modulator of ORIC run-down.

### Magnesium affects ATP-induced changes in ORIC dynamics

**Figure 2:**
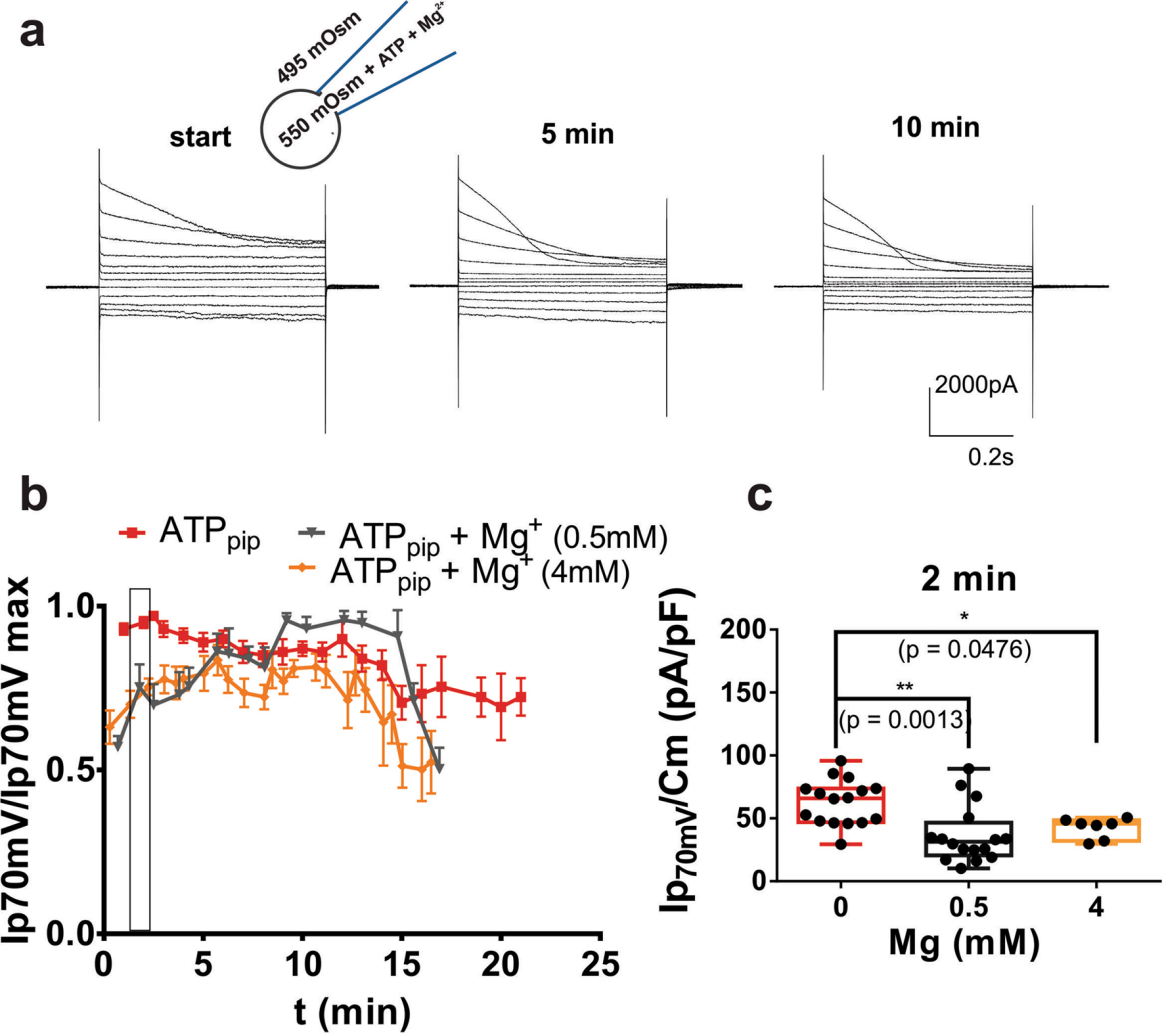
Mg^2+^ + ATP effect on ORIC run-down. (**a**) Representative recordings of ORIC voltage family with 4 mM MgATP included in the pipette in response to standard voltage protocol shown in Fig. 1a. Scheme depicting the extracellular and pipette conditions relevant for the recordings is shown above the recording at the start of the experiment. (**b**) Time dependence of the Ip70 mV, normalized to maximum value for each CD, ATP_pip_, (4 mM Mg^2+^ + ATP)_pip_ and (0.5 mM Mg^2+^ + ATP)_pip_. Notation at the top of the graph shows concentrations of free ATP and free Mg^2+^. Mean ± SE, rectangle marks the time point analyzed in graph in (**c**). n = 30 (ATP), 19 (4 mM Mg^2+^ + ATP) and 10 (0.5 mM Mg^2+^ + ATP). (**c**) Box and whisker plots enclosed by the 25th and 75th percentile range, median line with whiskers extending minimal to maximal value, all points shown. Peak current densities at + 70 mV, at 2 min after the whole cell break in, for group without Mg^2+^ (0 Mg^2+^  + ATP)_pip_, low Mg^2+^ (0.5 mM Mg^2+^  + ATP)_pip_ group and high Mg^2+^ (4 mM Mg^2+^  + ATP)_pip_ group. ANOVA with multiple comparisons to 0 Mg^2+^  + ATP group and Holm-Sidac correction. n = 22 (0 Mg^2+^  + ATP)_pip_, 16 (0.5 Mg^2+^  + ATP)_pip_ and 7 (4 Mg^2+^  + ATP)_pip.._

Next, we tested if Mg^2+^ ion, known to form a complex with ATP and influence interaction of ATP with some ATP-regulated channels^23^, has an effect on ORIC in the presence of ATP. As shown in Fig. 2a,b, ATP-mediated stabilization of ORIC is mildly negatively regulated by Mg^2+^, since time course of Ip70mV, normalized to maximal value, shows that ORIC activation is slowed down in (Mg^2+^ + ATP)_pip_. Comparison of ORIC density at 2 min (Fig. 2c) shows that, with (Mg^2+^ + ATP)_pip_, the current density is reduced at the early times. On the other hand, (Mg^2+^ + ATP)_pip_ is not an inhibitor of ORIC, since, after 5 min or later, amplitude of ORIC in (Mg^2+^ + ATP)_pip_ was not different from amplitude with ATP_pip_, (without Mg^2+^) (Fig. 2b). Both experimental groups with (Mg^2+^ + ATP)_pip_ had similar values of current density, showing that Mg^2+^ + ATP complex is probably the main form of Mg^2+^ exerting the effect. We conclude that from the finding that in the low Mg^2+^ group (60.6 µM free Mg^2+^, out of 0.5 mM total Mg^2+^), effect on ORIC persisted and was not different than effect of high Mg^2+^ (2.1 mM free Mg^2+^, out of 4 mM total Mg^2+^).

### ATP and AMP PCP cause a shift of voltage dependence of inactivating fraction of ORIC

**Figure 3:**
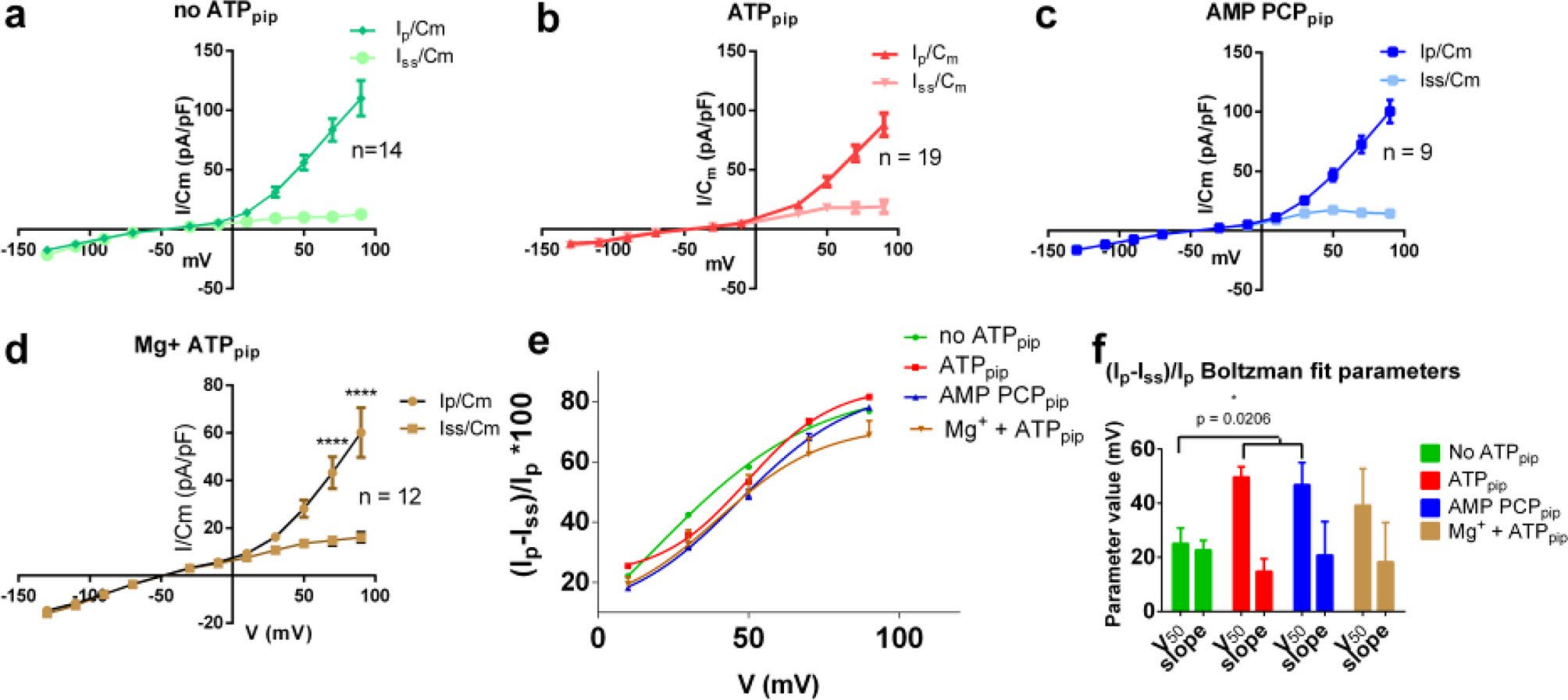
Voltage dependent properties of ORIC and effect of ATP. (**a**–**d**) IV curves of peak (Ip) and steady state (Iss) current density measured 2—3 min after break-in. Mean ± SE. (**a**) no ATP; (**b**) 2 mM ATP; (**c**) 2 mM AMP PCP; (**d**) 4 mM Mg + 2 mM ATP. IV dependence of ORIC steady-state current is unchanged by Mg^2+^, while ORIC peak current is significantly smaller than in other groups shown in (**a**–**c**). Two way ANOVA with Holm–Sidac correction; (**e**) Fraction of inactivating current (FIC) with Boltzmann fit curves of all groups shown in (**a**–**d**); (**f**) Parameters of Boltzmann fits (V_50_ and slope = RT/z_d_F) both in mV obtained from the curves in the graph in e, showing that ATP and AMP PCP significantly shift V_50_ of voltage dependency of FIC towards more depolarized potentials. Best fit values and SE of fit. Reported statistical differences obtained by comparison of Boltzmann fits extra-sum-of-squares F-test.

In order to test if stabilizing effect of ATP on ORIC is connected to the change in voltage-dependent current properties or to the increase of whole-cell current amplitude, we compared initial current density of all experimental groups after full two minutes post break-in. The current–voltage dependency of ORIC was unchanged in ATP_pip_ and AMP PCP_pip_ (Fig. 3a–c). Boltzmann fit parameters were similar in all groups, with V_50_ (in mV) = 61 ± 17 (no ATP_pip_), 68 ± 21 (ATP_pip_) and 69 ± 18 (AMP PCP_pip_), while number of the gating charges z_d_ was 1. Current density Ip70mV/Cm (mean ± SD in pA/pF) was 83.6 ± 35.3 (no ATP_pip_), 64.0 ± 26.0 (ATP_pip_) and 72.6 ± 21.3 (AMP PCP_pip_). Boltzmann fit parameters in Mg^2+^ + ATP were not statistically different from the corresponding parameters obtained for other groups.

It appears that ATP-binding effect is not mediated by activation of more channels, as that would be evident in an increased average current density of ORIC. As expected the effect of (Mg^2+^ + ATP)_pip_, due to Mg^2+^ + ATP-induced slow and incomplete activation at this time point, is a reduction in peak current density at highest depolarized potentials (+ 70 and + 90 mV, p = 0.0004 and p < 0.0001, respectively, compared to ATP_pip_ and for both V_h_ p < 0.0001, compared to no ATP), while steady-state current density remained unchanged (Fig. 3d).

Proportion of the current that gets inactivated by depolarizing voltage pulse, measured as Fraction of Inactivating Current (FIC, calculated as (Ip − Iss)/Ip), was modified in ATP_pip_ and AMP-PCP_pip_. The Boltzmann equation was fitted to the voltage dependence of FIC (Fig. 3e) and obtained fit values (half inactivation voltage V_50_ and the slope of the curves) are compared to no ATP_pip_ (Fig. 3f). While FIC showed similar voltage dependence in ATP_pip_ and AMP-PCP_pip_, either treatment shifted the inactivation towards the depolarized potentials. Half-inactivation voltage (V_50_) increased from 8 ± 2 mV in no ATP_pip_, to 35 ± 4 mV with ATP_pip_, and 36 ± 3 mV in AMP PCP_pip_. (Fig. 3f), extra sum-of-squares F-test comparing Boltzmann fit parameters, p < 0.0001. A similar FIC slope (24 ± 2), for all three treatments suggests that neither ATP or non-hydrolizable ATP-binding affected the voltage sensitivity of inactivation; Rather, ATP increased the proportion of non-inactivated current at any potential. Boltzmann fit of the (Mg^2+^ + ATP)_pip_ FIC was not statistically different (parameter values obtained: V_50_ = 39 ± 11 mV, slope = 18 ± 14) than ATP_pip_ or AMP PCP_pip_.

### Cytoplasmic droplets are metabolically active and rate of ORIC inactivation is sensitive to presence of ATP in the droplet

**Figure 4:**
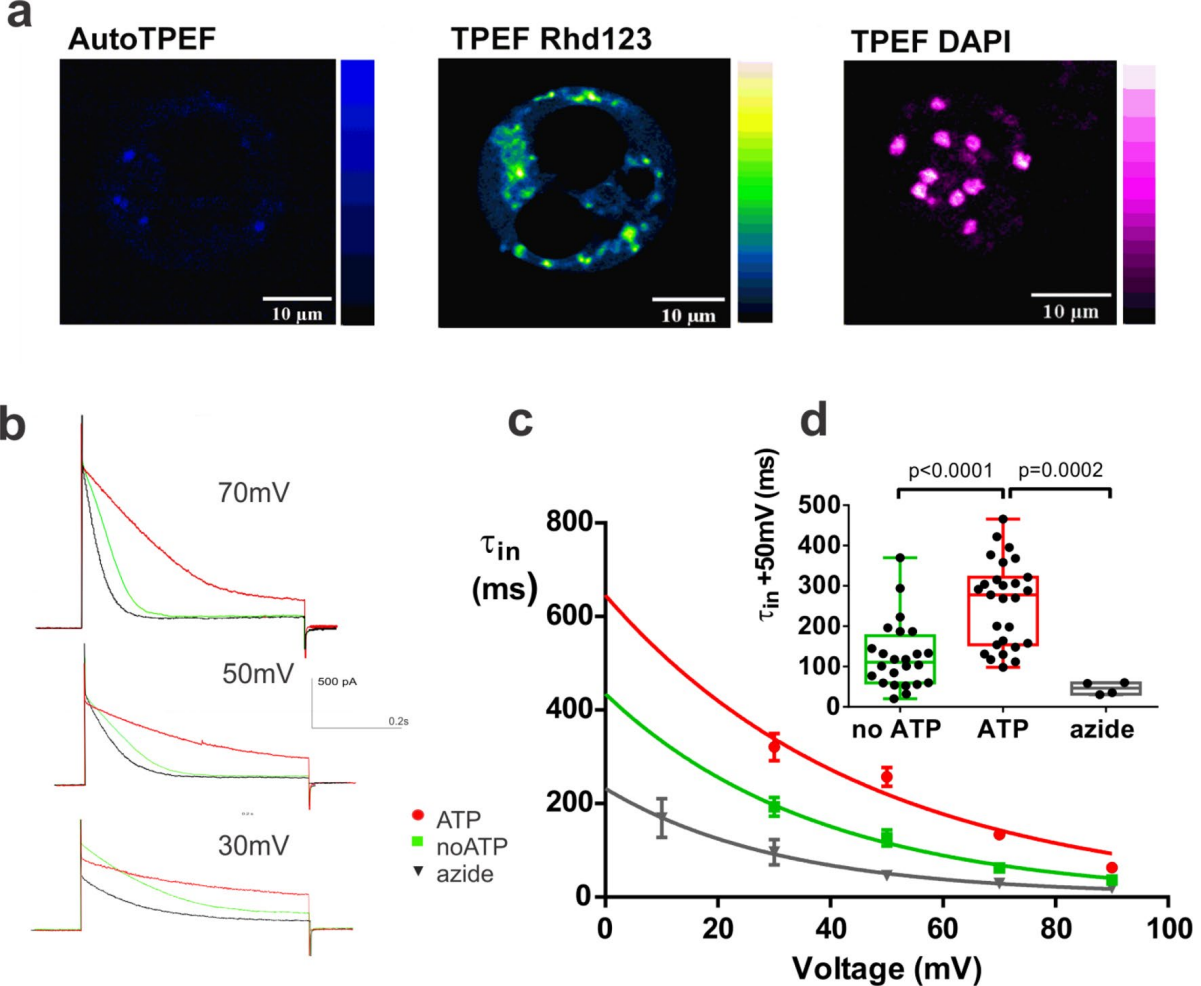
Cytoplasmic droplets are metabolically active: they contain functional mitochondria and ORIC is sensitive to preincubation of droplets with metabolic inhibitor. (**a**) Left: Autofluorescence NAD(P)H signal, showing bright spots in cytoplasm (AutoTPEF). P at sample = 3.8 mW, 730 nm, filter VIS + 479/40. Middle: Rhodamine123-stained cytoplasmic droplet showing active hyperpolarized mitochondria as bright structures (TPEF Rhd123). P at sample = 3.5 mW, 800 nm, filter VIS + 530/43. Right: DAPI staining reveals presence of multiple nuclei distributed throughout the cytoplasmic droplet (TPEF DAPI). P at sample = 5.6 mW, 730 nm, filter VIS + 479/40. (**b**) Aligned current responses to + 70 mV, + 50 mV and + 30 mV steps (at 2 min) in control conditions (no ATP_pip_), with ATP (ATP_pip_) and after preincubation in sodium azide (azide + no ATP_pip_). (**c**) Voltage dependency of τ_in_ (mean ± S.E); (**d**) box and whisker plot of τ_in_ at 50 mV, all points shown (right panel) for the same experimental groups as shown in (**a**). ANOVA, multiple comparisons with Holm–Sidac correction of τ_in_ at 50 mV, n(no ATP) = 24, n(ATP) = 27, n(azide) = 4.

CDs represent dynamic membrane-enclosed systems, with densely granulated cytoplasm in motion, and bearing several prominent empty regions, presumably of vacuolar origin. Imaging of CDs with settings enabling the detection of NAD(P)H auto fluorescence reveals faint signal in the cytoplasm with several prominent bright spots (Fig. 4a left). It is possible that spots of high NAD(P)H signal are mitochondria. In order to confirm that mitochondria are present in CDs, we stained CDs with rhodamine 123 (Rhd123), the dye that fluoresces in healthy hyperpolarized mitochondria^24^ (Fig. 4a middle). Numerous bright spots representing functional mitochondria were revealed by Rhd123 staining. Round structures of approximately 2 µm diameter in DAPI stained CDs image (Fig. 4a right) are nuclei, confirming that CDs are dynamic and complex membrane enclosed systems.

Since CDs have functional mitochondria, it is not surprising that the ORIC in some CDs exhibited similar properties to the ATP_pip_ group in recordings made immediately after whole-cell break-in without ATP addition. The V_50_ of the inactivating current FIC ((Ip − Iss)/Ip) at the beginning of the recordings was 19 ± 8 mV (n = 6), which was not statistically different from the fit of the FIC in the ATP_pip_ group. After standard two-minute dialysis, the V_50_ of the FIC shifted for the same CDs toward hyperpolarized values, to 5 ± 6 mV (Supplementary Fig. 2, left). The increased within-group variability (Supplementary Fig. 2, right) in no ATP_pip_ group before substantial dialysis suggested that the CDs likely had different initial amounts of ATP available for ORIC modulation. The different ATP content of the CDs reflected in the ORIC properties suggests that not all CDs are equally metabolically active.

We have previously described that ORIC inactivation appears to be voltage dependent and that the rate of inactivation appears to increase over the course of the recording as the run-down progresses. In order to test the effects of ATP on these processes, we compared the speed of ORIC inactivation with and without ATP, quantified as the time constant of inactivation (τ_in_) obtained from the exponential function fitted to decaying part of current recordings (Fig. 4b). τ_in_ at each potential is notably larger in ATP_pip_ (Fig. 4c). Difference is statistically significant (p < 0.0001) at + 50 mV depolarizing stimuli, with values of τ_in_ (+ 50 mV) in ms (mean ± SD): No ATP_pip_ = 126 ± 83, n = 27; ATP_pip_ 257 ± 105, n = 24 (Fig. 4d). Accordingly, 2 mM azide, used to inhibit respiration and reduce basal ATP content of CDs, reduced τ_in_ (+ 50 mV) = 46 ± 15, n = 4, values significantly different than ATP_pip_ (Fig. 4d). However, the rate of change of τ_in_ with voltage is similar in all groups, approximately 0.26 s with every 10 mV. We conclude that, in the presence of ATP, ORIC inactivation is slower (τ_in_ is larger), while the rate of change of τ_in_ with voltage is unchanged. Since ORIC inactivation is a depolarization-induced process, it seems that ATP shifts the balance between activated and inactivated current without affecting the voltage dependency of inactivation. In order to achieve the reduction of inactivation speed without change in voltage sensitivity, ATP binding would have to stabilize a state of the underlining channel that is not affected by voltage, possibly some open state.

### Recovery from inactivation is voltage-dependent, and incomplete at depolarized potentials

**Figure 5:**
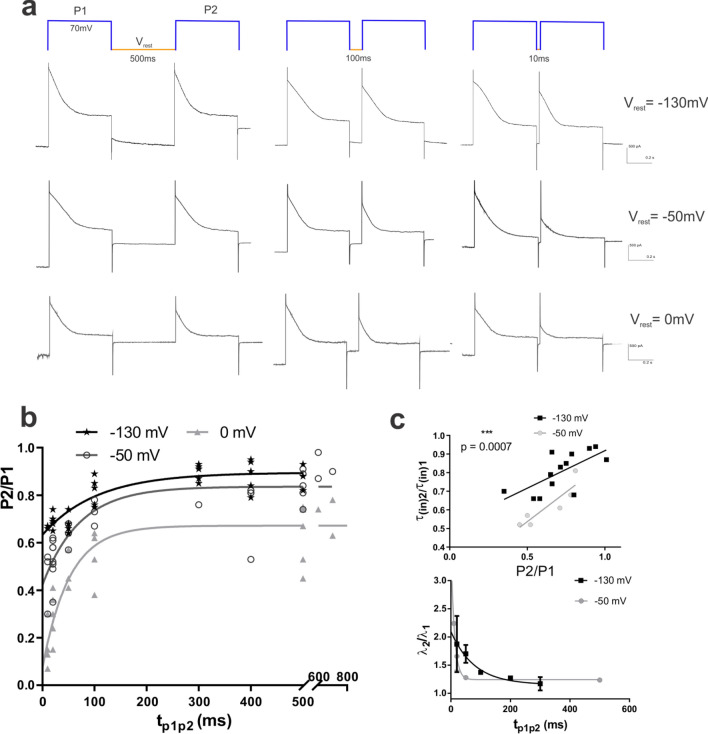
Recovery from inactivation of ORIC with ATP_pip_. (**a**) Representative recordings of ORIC responses to P2/P1 protocols for measuring recovery from inactivation upon depolarization step (P1). The protocol scheme with 3 exemplary recovery times t_p1p2_ (500 ms, 100 ms and 10 ms) between inactivating step P1 and test step P2 is depicted in the top row. Holding potential during recovery period, V_rest_, is indicated on the far right end of each row with current recordings (− 130 mV, − 50 mV, and 0 mV). (**b**) Dependency of recovered test amplitude P2, normalized to start amplitude P1 (P2/P1) on time allowed for recovery t_p1p2_. Plot of all points shown. Points with different V_rest_ are presented as separate series. One component exponential fits of the P2/P1 vs t_p1p2_ are shown for each V_rest_ series. (**c**) Decrease of time constant of current inactivation τ_in_ in recordings obtained with P2/P1 protocol corresponds tightly to the decrease of amplitude. Top: correlation of decrease of time constant of inactivation of ORIC current (τ_in2_/τ_in1_) with the decrease of the amplitude of inactivating component of current (P2/P1), p = 0.0007; Bottom: P2/P1 inactivation rate ratio λ_(in)2_/λ_(in)1_ plotted as a function of recovery time duration (t_p1p2_) at two V_rest_ potentials (− 130 and − 50 mV). Data is fitted with the exponential function with τ_(− 130 mV)_ = 79 ms and τ_(− 50 mV)_ = 11 ms.

Once we removed the time-dependent increase of inactivation, detected as run-down, by adding ATP, we could analyze depolarization-induced voltage dependent inactivation of ORIC. The P2/P1 protocols were used with varied time of recovery, t_p1p2_ (10–700 ms) at different resting potentials, V_rest_ (− 130 mV, − 50 mV, and 0 mV) (Fig. 5a). We compared values of Ip − Iss to obtain time and voltage dependence of ORIC recovery from inactivation, un-obscured with any changes of Iss. Resulting recovery curves are depicted in Fig. 5b. Fitting the exponential function to the data showed that the recovery from inactivation takes more time at hyperpolarized potentials (τ_rec_ at 130 mV was 78–58 ms, while at 0 mV, τ_rec_ was 34–67 ms). At the V_rest_ 0 mV, after longest tested recovery time, ORIC inactivation recovery reached the plateau of only 67 ± 3%. Compared to the plateau recovery obtained at − 50 and − 130 mV (84 ± 3% and 90 ± 2%, respectively), maximal recovery at 0 mV is incomplete during 700 ms, the maximal time we used for P2/P1 protocol. Extra sum-of squares model comparison shows that plateau values differ significantly (p < 0.0001) between three curves obtained at tested V_rest_.

It is clear from Fig. 5b that recovery from inactivation is dominated by the pool available at the start of the recovery (the portion of available current at the minimal t_p1p2_), resulting in significantly different value of recovered current at longest time (plateau). This result, taking into consideration that the rate of recovery is not faster at hyperpolarized V_rest_, can be explained either by: (1) very fast voltage dependent recovery process that would be completed in less than 10 ms at hyperpolarizing V_rest_; (2) the voltage dependent inactivation process that brings much more channels to inactivated state at 0 mV than at hyperpolarized potentials. Correlation between the amplitude drop and an increase of inactivation speed during t_p1p2_ suggests that these two processes, current amplitude decrease and speeding-up of current inactivation during recovery time, are tightly connected (p = 0.0007) (Fig. 5c, upper graph). Our working model, based on all the data is that, without ATP, ORIC does not recover from inactivation efficiently, resulting in the current run-down. With ATP_pip_ present, at hyperpolarized potentials ORIC very quickly reaches almost complete recovery from inactivation. Speeding-up of current inactivation, detected as a decrease of inactivation time constant, is interconnected with the extent of recovery from inactivation (Fig. 5c, bottom graph). After 10 ms of recovery at − 50 mV, ORIC inactivates with 124% larger rate λ_in,_ compared to λ_in_ during P1. When more time is allowed for recovery, for instance 50 ms of recovery at − 50 mV, ORIC inactivates with only 30% larger rate λ_in_ than during P1. It should be noted that, after the same time of recovery (50 ms) at − 130 mV, inactivation speeding-up is larger than at − 50 mV: ORIC inactivates with 70% larger rate λ_in_ than during P1. Such ORIC behavior suggests that during time of recovery, there is a net-effect influencing inactivation rate, making current inactivation faster when current recovery occurs at more hyperpolarized potential.

### Pharmacological properties of ORIC

**Figure 6:**
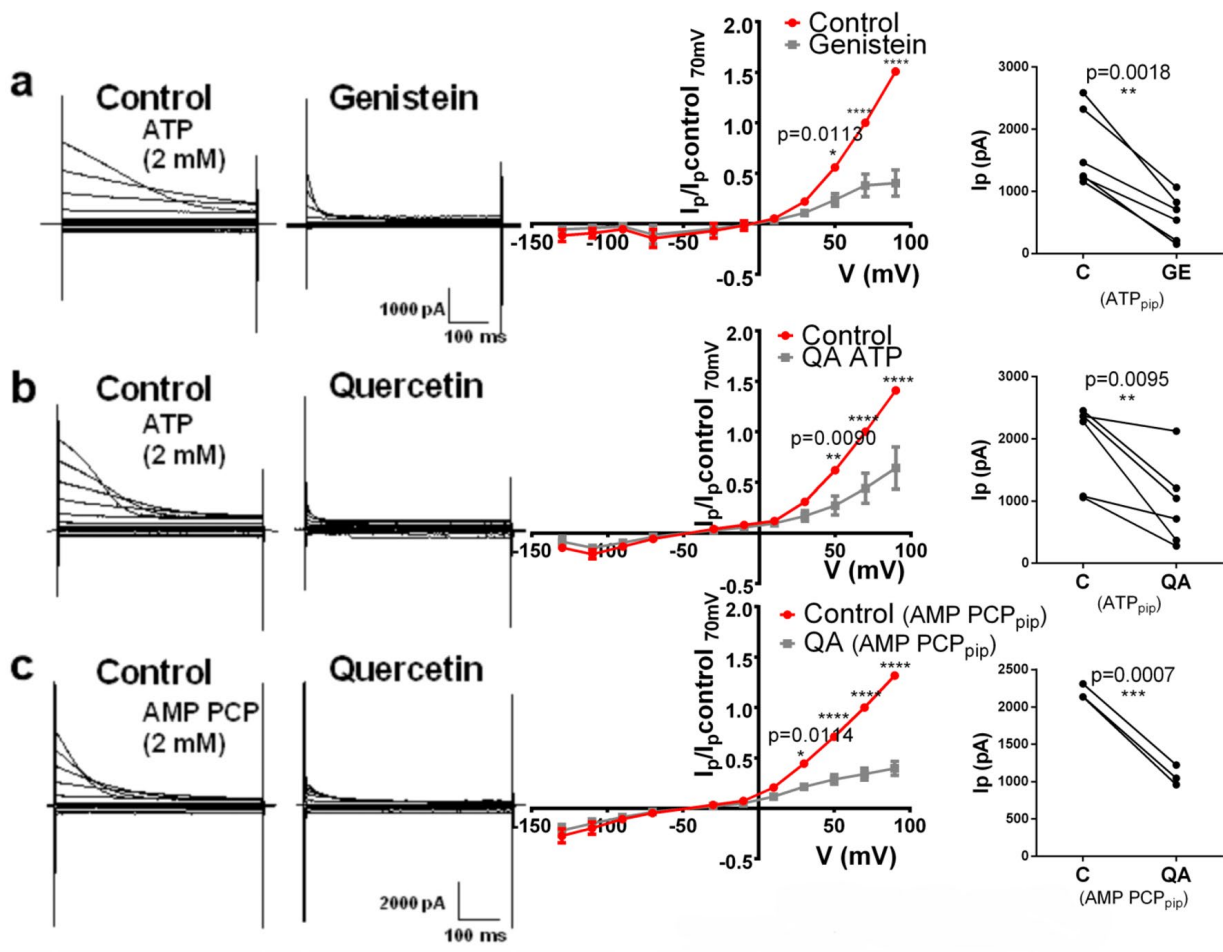
Flavonoids block ORIC. Left panels (**a**–**c**) show representative recordings before (annotated as Control with corresponding pipette content) and after (3 min incubation after addition) of extracellular addition of flavonoids (100 µM): (**a**) Genistein with 2 mM ATP_pip_, (**b**) Quercetin with 2 mM ATP_pip,_ (**c**) Quercetin with 2 mM AMP PCP_pip_. Standard voltage protocol as in Fig. 1a was used to generate current families depicted. Middle panels show corresponding IV curves of peak current normalized to control value at + 70 mV: red symbols and connecting lines for control and gray for treatment. Repeated measures two way ANOVA with Holm–Sidac correction. Far right panels show corresponding plots of Ip70mV for paired control and treatment recordings in all experiments. ANOVA with Holm–Sidac correction, p < 0.001.

To explore the pharmacological properties of ORIC ATP dependency, we used flavonoid compounds, known to block kinases and channels with ATP binding site. Genistein and quercetin (at 100 µM) both blocked ORIC with ATP_pip_ (Fig. 6a,b). Flavonoid inhibitory effect on Ip70mV of ORIC was significant, as in genistein ORIC was reduced to 33.3 ± 15.4% of control value (mean ± SD), p = 0.018 (n = 5); Quercetin decreased ORIC Ip70mV amplitude to 48.5 ± 26.9% of control current, p = 0.0095. Inhibition of ORIC was significant at all potentials where inactivating current was prominent, as evident in IV curves (Fig. 6a,b, middle panels). Using quercetin (at same concentration), we explored whether flavonoid block of ORIC persists when ATP is replaced equimolarly with AMP PCP (Fig. 6c). Quercetin inhibited ORIC Ip70mV by 50 ± 1%, p = 0.0007 (Fig. 6c, right), demonstrating that for inhibition of ORIC by flavonoids, intracellular ATP exerts its effect in a manner not requiring ATP hydrolysis. The fraction of quercetin-inhibited current with AMP PCP_pip_ was not statistically different from that with ATP_pip_. IV curves in quercetin with AMP PCP_pip_ (Fig. 6c, middle panel) showed inhibition at all inactivating depolarizing potentials, similar to ATP_pip_.

**Figure 7:**
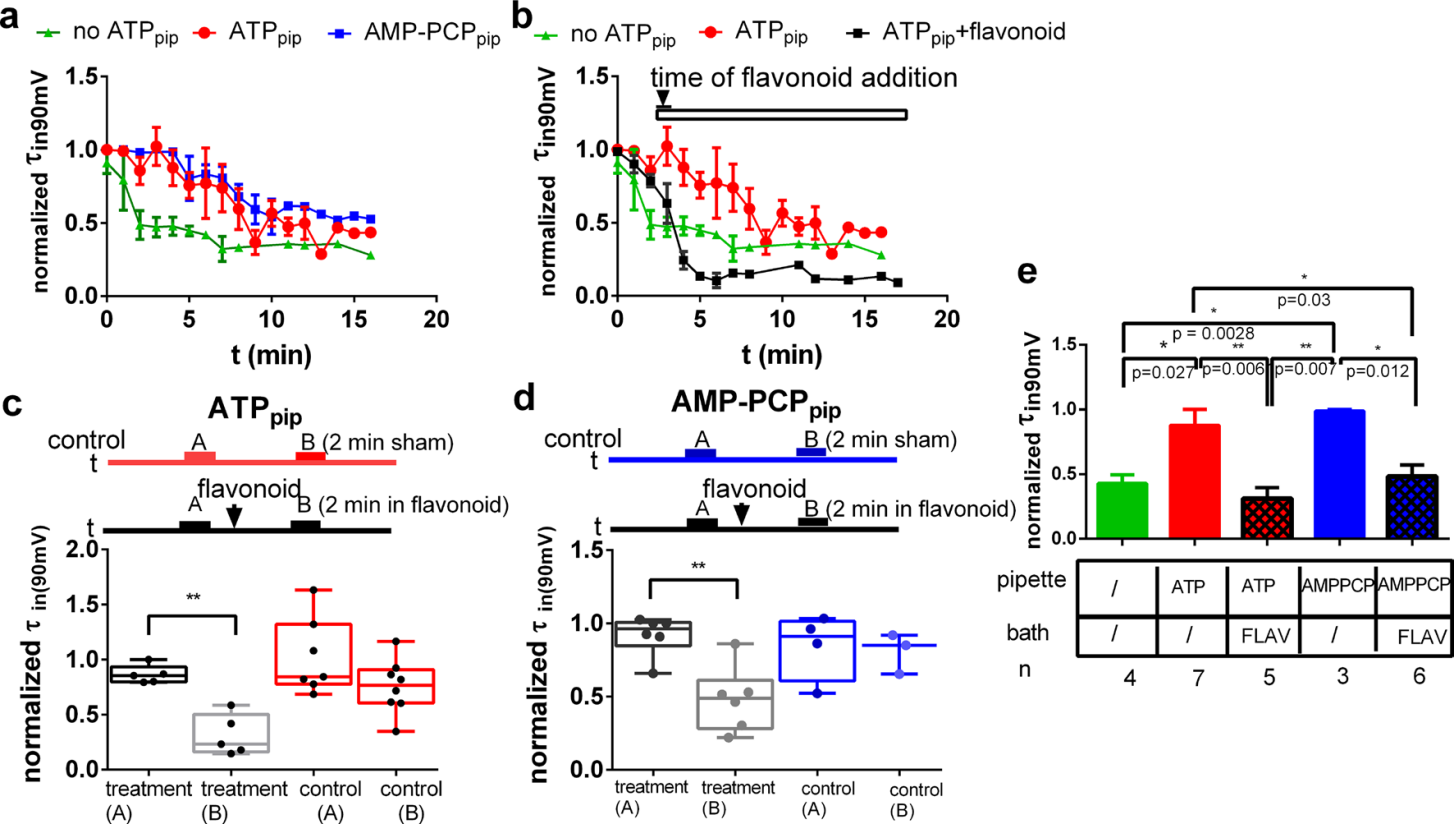
Blocking of ORIC by flavonoids leads to a decrease in the time constant of current inactivation to values comparable to those obtained in the absence of ATP. (**a**) Time dependence of ORIC inactivation time constant (τ_in_) at voltage stimulation of 90 mV, normalized to the maximum value in that droplet, measured in three separate groups: 2 mM ATP_pip_(red), 2 mM AMP PCP_pip_ (blue) and no ATP_pip_(green) (**b**) The decrease in ORIC τ_in_, upon flavonoid addition plotted along with the same curves for time-dependent change in τ_in_ in conditions with and without ATP_pip._ (**c**) and (**d**) Box and whisker plots showing the difference between normalized τ_in_ for time points at two minutes after flavonoid addition and the same time point in the control experiments with (**c**) ATP_pip_ and (**d**) AMP PCP_pip_, the schemes above the plots emphasize that the same time span between the τ_in_ measurements was used in control (sham added) and flavonoids addition experiments. One way ANOVA, without matching, for selected pairs with Holm–Sidac correction. (**e**) Bar graph showing the comparison of τ_in_ for the same time points for all measurement conditions (5 min from the start of recording, 2 min in flavonoids). One way ANOVA with Holm–Sidac correction.

Next, we measured if flavonoids increase ORIC inactivation (Fig. 7), which would be expected if their binding prevents the effect of ATP. In no ATP_pip_, during current run-down (Fig. 7a), τ_in_ decreased by 50% after 3—4 min and asymptotically decreased to 30% after 7 min. This result seems to indicate the same process as shown in Fig. 5c, where τ_in_ change correlated with the amplitude decrease in the P1/P2 protocol. In ATP_pip_ or AMP PCP_pip_, τ_in_ is only slightly changed in the first 10 min, and afterwards is on average decreased to around 50% of the starting value. However, when flavonoids are added, a sudden drop of τ_in_ to less than 20% of the initial value is evident, lower than in no ATP_pip_ group (Fig. 7b). Next, we analyzed the same fixed time in both control experiments and experiments in which flavonoids were added (both with ATP_pip_ present). Statistical comparison (Fig. 7c,d) demonstrates that during 2 min of incubation of flavonoids, τ_in_ is significantly reduced, while without flavonoids addition, during same period τ_in_ is unchanged. Statistical comparison of normalized τ_in_ at the same time point (Fig. 7e) for all combinations tested shows that τ_in_ in flavonoids (in ATP_pip_ or AMP PCP_pip_) is undistinguishable from τ_in_ without ATP. It seems that, at least in part, flavonoid inhibitory effect on ORIC is mediated by the process that renders the current unable to be stabilized by ATP binding.

## Discussion

This work shows that ATP modulates and stabilizes ORIC, osmotically activated anionic current in sporangiophore-derived cytoplasmic droplets, the model of filamentous fungi plasma membrane. ATP binding, not hydrolysis, is required for maintenance of ORIC activity, attained through shifting the voltage dependency of inactivation towards more depolarized potentials. We found ATP-induced slowing down of ORIC inactivation as evidenced by: reduced current run-down, increased time constant of inactivation τ_in_ and slowed or completely abrogated decreasing of τ_in_ during prolonged recordings. We showed that active mitochondria are present in cytoplasmic droplets, and that inhibition of respiration by azide speeds-up ORIC inactivation, effectively acting as a current inhibitor.

The most striking feature of ORIC modulation by ATP is the postponing of the current rundown, previously shown to be characteristic for ORIC recordings without ATP addition to pipette^1^. This modulation does not require ATP hydrolysis, since AMP PCP, nonhydrolisable highly efficient ATP analogue of pre-hydrolytic state with slightly rigid structure^25^, delays ORIC inactivation as effectively as ATP. Substituting AMP PCP for ATP did not make a difference in any ORIC property tested: run-down reduction, τ_in_ and the speed of τ_in_ decline. In the case of VRAC, vertebrate osmotically activated anion channel with extensive biophysical similarity to ORIC, ATP exerts a stabilizing effect without hydrolysis as well^21,26^. Among known fungi channels, TOK channel in yeast requires ATP presence to avoid inactivation by run-down^27^. Other examples of ion channel modulation by ATP binding without hydrolysis exist in literature^25,28^. Typical examples are the ATP-sensitive K^+^ (K_ATP_) channels, regulated by ATP that acts as an allosteric modulator^22,29^. Activity of K_ATP_ channels is a balance between two opposing effects: ATP and ADP promote closed state of a channel (Mg^2+^-independently) by binding to inhibitory sites, while in the presence of Mg^2+^, ADP and ATP also stimulate channel activation by binding to another site^23^. Reminiscent to this example of Mg-ATP specific interaction that is different than ATP interaction with a channel, it seems that in the case of ORIC, Mg^2+^ exerts its effect on current only in the presence of ATP. Mild current inhibition by intracellular Mg^2+^, along with unchanged voltage-dependent properties in Mg^2+^, are reported for VRAC^30^, while for ORIC, we found that Mg^2+^ in the presence of ATP does not inhibit the current, and that main Mg^2+^ effect, completely dependent on presence of ATP, is slowing down the activation of current with no change in voltage dependency. It should be noted that, although in our study Mg^2+^ was used as a tool to examine mechanistic relationship between ATP and ORIC, free Mg^2+^ in physiological conditions is considered to be kept at homeostatic level. Tight regulation of free Mg^2+^ is linked to the paramount importance of Mg^2+^as a cofactor for various cellular enzymes and proteins and other essential functions^31^. Free Mg^2+^ in mammalian cells is considered to be maintained at fairly constant value around 1 mM or bellow^32^ and in the yeast cytoplasm it is at similar value (1.3 mM)^33^ while the depletion of Mg^2+^ induces changes in the yeast cell cycle^34^. Therefore, we cannot exclude the possibility that ORIC could be modulated by the transient Mg^2+^ fluxes, for instance due to exposure to depleting environmental conditions, or on the other hand, very high [Mg^2+^] environment as it is known to occur in filamentous fungi^35^. Unlike ATP, GTP does not slow down inactivation of ORIC, as shown here by a lack of reduction of run-down, suggesting that ORIC is not equally modulated by all nucleotide triphosphates.

The interconnectedness of ORIC run-down and current inactivation process, defined as depolarization-induced inactivation from the open state and described with τ_in_, is a plausible explanation of several obtained results: run-down is accompanied by speeding-up of current inactivation; ATP, or nonhydrolyzable ATP analogue, reduces both phenomena; flavonoids have dramatic effect on both processes. Similar current behavior, with run-down tightly correlated with voltage-dependent inactivation, is described in calcium voltage-activated channels, for instance^36^. It is possible that other processes of inactivation during run-down occur, from closed state, as well as dialysis-driven wash-out of regulatory components that contributes to current amplitude decline^37^.

We could measure the speed of ORIC recovery only in the presence of ATP, due to otherwise dominating current run-down. In the presence of ATP, current recovery from inactivation was incomplete at depolarized potentials, potentially giving rise to accumulation of ORIC in inactivated state, as shown by the differences in plateau of recovery curves at different voltages. In contrast to described behavior of VRAC^38^, the measured speed of the ORIC recovery from inactivation was voltage independent, or even slightly faster at depolarized potentials.

Based on our measurements of changes in inactivation dynamics during the flavonoid block, it seems that the flavonoids block ORIC by inducing changes that correspond to disruptions of ORIC modulation by ATP. Flavonoids, formerly considered to be specific inhibitors of protein/lipid kinases^39^, are also known to bind to a number of binding sites on various proteins^40^, due to their partially flexible structure. Mammalian cell-wide search showed they have a wide range of specific target proteins, belonging to approximately eight structural folds^41^. They can act as competitive antagonists to ATP at the ion channel itself, as studies on cardiovascular channels imply^42^, or at channel accessory proteins. Shift of voltage dependence of current inactivation in the hyperpolarizing direction by genistein has been reported for transient outward K^+^ current in the heart, mediated by voltage-gated Kv4.3^43^. Numerous ion channels are known to be modulated by flavonoids: cAMP-induced delayed rectifier K current^44^, Ca^2+^^45^ and GABA_A_ channels^46^, to name a few. Some studies suggest that genistein effect on ion channels is due to alteration of mechanical properties of membrane bilayer, as suggested by work on gramicidin A^47^. In other cases, the flavonoid effect on ion channel is specific and mediated by direct binding to channel protein, as demonstrated for ASIC^48^. A large number of flavonoids have been shown to inhibit VRAC^49^, with flavonol quercetin and the isoflavone genistein, which we tested at ORIC, among its most potent inhibitors. Flavonoids inhibit ORIC, even with ATP is substituted with AMP PCP, and in the same manner (reduction of current amplitude and speed-up of inactivation, measured by τ_in_). We propose that flavonoid binding precludes ATP or its analogue from exerting its stabilizing effect. Similar findings were reported for VRAC^50^.

We also found that cytoplasmic droplets (CDs) varied in the ORIC properties at the time of start of the recording, presumably due to varied amount of ATP present in the droplet at the time of whole-cell entry. After several minutes of dialysis with an ATP-free solution, resulting in bringing all the CDs to the same low ATP content, we found as expected, a decrease of variability of all measured parameters. Since we used third-minute-of-dialysis data for all comparisons, we are confident that observed changes are indeed the result of presence or absence of ATP in dialysis solution.

We have confirmed the plasma membrane-nature of CD membrane: previously, our research group has shown that CDs can regenerate the cell wall^1^, while here we showed fast and robust depolarization of CD membrane by vanadate (shown in “Materials and methods” section), known to specifically block the plasma membrane proton pump of fungi^51^. Fluorescence imaging demonstrated that the cytoplasmic droplets are dynamic structures containing a large number of mitochondria and, because they originate from a cenocytic sporangiophore, a large number of nuclei. Therefore, an ion channel in the CD membrane has a similar intracellular environment as in an intact fungus, and the CD current recordings are obtained in physiologically relevant context. Studies of fungal ion channels are mostly executed by heterologous expression in yeast^52^ and oocytes, models that potentially differ from the physiological environment of a filamentous fungi cell. On the other hand, our native membrane model system, CDs, does not offer information on molecular identity of the ion channel underlining ORIC.

The search for VRAC-homologue in available DNA and protein sequence databases for *Phycomyces blakesleeanus* and other filamentous fungi yielded no resulting sequences. This negative result, the absence of sequences with substantial homology to LRRC8A-D, suggests that ORIC-mediating channel is not VRAC homologue on the level of primary sequence. This is expected since VRAC mediating ion channels are not found outside vertebrate clade^53^. We recently explored and characterized additional biophysical similarities between ORIC and VRAC^54^, while we also identified several important distinctions of ORIC in comparison to VRAC: in single channel conductance, reversed dependence of inactivation speed and permeability^1,54^. All available data point to conclusion that ORIC could be a functional homologue of VRAC without bearing extensive sequence homology.

To the best of our knowledge, so far there have been no reports on a fungi-derived ion current with any similarity to ORIC. Although it is possible that the underlining channel is identified, but drastically changed properties in non-native context have rendered it to be unrecognizable, it is probably more likely that, due to the low sequence similarity to animal channels, proteins that constitute a membrane ion channel that mediates ORIC have not been identified yet.

Several anion channels from filamentous fungi have been characterized and identified by heterologous expression. AnBEST from *A. nidulans*, anion efflux channel activated by alleviated cytosolic Ca^2+^, was recorded by expression in yeast, and confirmed to be located in *A. nidulans* plasma membrane by GFP labeling^52^. Member of anion channel ClC superfamily from *A. nidulans*, involved in copper homeostasis, is likely expressed in endomembranes. Others have been described in native membrane of protoplasts released after laser ablation of cell wall: 43pS anion efflux channel in *A. niger*^55^, and unidentified channels in *N. crassa*^56^. In CDs, our group described malate-sensitive 10pS depolarization activated outwardly rectified anionic current^11^.

Osmotically activated current like ORIC could be involved in some process linked to the growth, since turgor is driving the growth process^57^, along with tip-end ion gradients^58^, while ORIC is present on the membrane obtained from the region actively growing. CD membrane potential is more depolarized than expected for filamentous fungi hypha^59^, in accordance with voltage–sensitive dye measurements from *Candida* demonstrating that growing parts of membrane are typically depolarized^60^. The role of ORIC-mediated anion transport is probably in anion efflux, since ORIC mediates exit of anions at potentials characteristic for filamentous fungi membrane^59,61^.

ORIC dependence on ATP points to its role as a metabolic sensor, in addition to its primary function in osmotic sensing. In filamentous fungi, the essential role of the hyperosmotic-response pathway in nutrient sensing and its direct connection to metabolism regulation has been described in *Neurospora*^62^. The inhibition of ORIC by pretreatment of CD with azide demonstrates the metabolic sensing role of ORIC, since, as described by our group^63^, sodium-azide is a potent blocker of cellular respiration in *P. blakesleeanus* in the concentration range used in this study*.* As a consequence of respiration block by azide, the ratio of core polyphosphates to inorganic phosphate (Pp/Pi) in *P. blakesleeanus* is reduced, showing the efficient reduction of metabolic activity. Pp/Pi is tightly connected to the oxidative phosphorylation chain and was previously shown to be a good indicator of metabolic state of *P. blakesleeanus*^63^. Inactivation of ORIC under the conditions of ATP scarcity is likely to have a protective role, since hypoosmotic conditions can signal lack of sugars in the environment. If it is beneficial to shut off ORIC during starvation, this current is probably not critical for basic survival mechanisms under conditions of prolonged starvation and osmotic stress.

In conclusion, ion channels, vital components of filamentous fungi signaling machinery in communication with their environment, are still relatively under-investigated, although they represent potential targets in biotechnologically and biomedically important organisms. We described ATP modulation of osmotically activated anion current in filamentous fungus *Phycomyces blakesleeanus*, of order Mucorales. Recently published World Health Organization (WHO) fungal priority pathogens list puts entire order Mucorales in high priority group for surveillance, research and development of new drug targets^64^. Further ORIC characterization, bringing to light the molecular identity and effect of its knockout on entire organism will potentially contribute to WHO goal, as well as to the better understanding of osmotic and metabolic responsiveness of filamentous fungi.

## Materials and methods

### Model system

Wild-type strain of fungus *Phycomyces blakesleeanus* (Burgeff) NRRL1555 (–) was grown on rich potato-dextrose-agar medium by standardized protocol^65^ in glass vials, in growth chamber with continuous overhead white light of 10 W/m^2^, at 20–23 °C and ca. 95% relative humidity, for 72 h to allow development of mycelium bearing sporangiophores in IVb stadium (with black sporangium). Mycelium has the capacity to produce new sporangiophores, after removal of used ones within 24 h, up to 3 times.

### Solutions and chemicals

Standard extracellular solution used (SE) contained (in mM): 65 KCl, 60 K-glutamate, 10 HEPES, 2 MgCl_2_, 1 CaCl_2_, sucrose, pH 7.1 (adjusted to 495–505 mOsm). Standard pipette solution (SP) for hypoosmotic activation of ORIC contained (mM): 65 KCl, 60 K-glutamate, 10 HEPES, 2 EGTA, 2 Na_2_ATP (Sigma Aldrich*)*, adjusted by sucrose to 550 mOsm, pH 7.1. Pipette solution for testing the spontaneous rundown of the current contained no ATP. In order to test the effect of nonhydrolisable ATP analogue, ATP was equimolarly substituted by AMP-PCP (adenylyl-(β,γ-methylene)-diphosphonate, tetralithium salt, Sigma Aldrich). For GTP effect measurements, we used sodium guanosine triphosphate (Sigma) concentration 2 mM, diluted in SP and applied as pipette solution. To study the effect of Mg^2+^ in presence of ATP, two concentrations of MgCl_2_ were used to modify the SP: 0.5 mM and 4 mM. Free Mg^2+^ concentrations were calculated using Ca^2+^/Mg^2+^/ATP/EGTA Calculator v1^66^ (https://somapp.ucdmc.ucdavis.edu/pharmacology/bers/maxchelator/CaMgATPEGTA-TS.htm). For current clamp recordings, extracellular solution with reduced ionic strength that resembles artificial pond water (APW) was used: 1 mM CaCl_2_, 2 mM MgCl_2_ 1 mM KCl, 489 mM sucrose. Measured pH was 6.0. Blocking agents were prepared as stock concentrations in DMSO (flavonoids) or extracellular solution (ATP), and diluted in microscopic chamber to the final concentration (100 µM of flavones genistein (Tokyo chemical industry*)* and querzetin (Sigma Aldrich), and a range of concentrations from 25 μM to 2 mM for ATP). A 200 mM stock solution of sodium-vanadate (Sigma Aldrich) was made in water and pH was adjusted to pH 10 with HCl. All stocks of blocking agents were stored at − 20 °C.

### Preparation of cytoplasmic droplets

**Figure 8:**
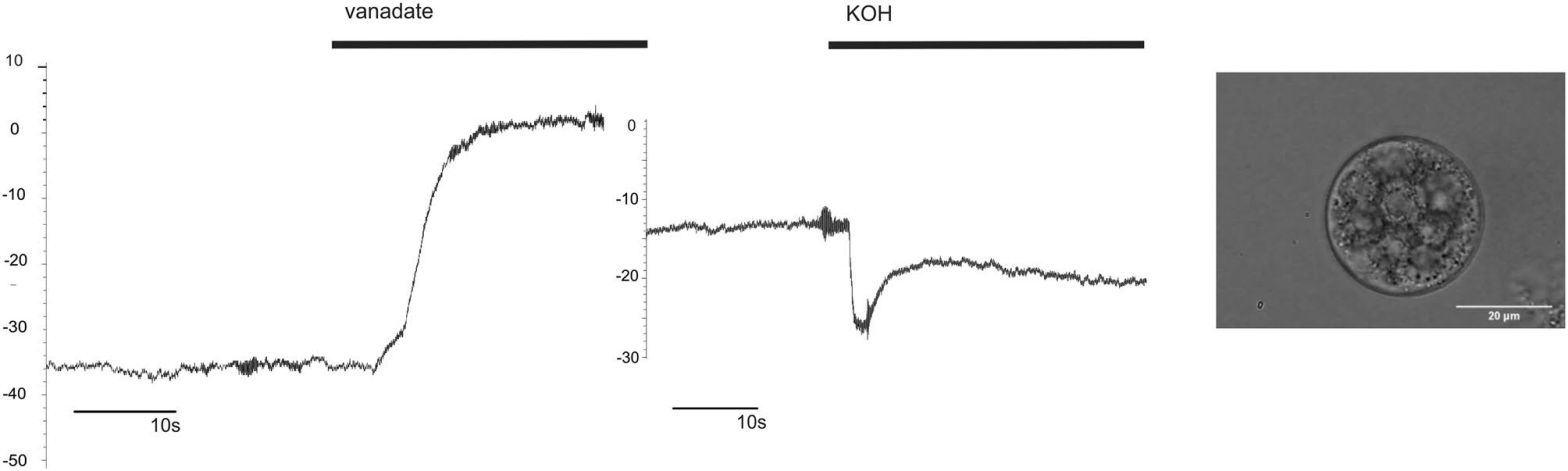
Vanadate depolarizes cytoplasmic droplet membrane, as expected for structures of plasma membrane origin. Left: Current clamp recording, with bar on top showing the time vanadate is present in the bath solution (n = 3). Right: Control current clamp recording of effect of KOH (in the amount corresponding to pH change induced by the addition of vanadate. Bar on top shows the time of KOH addition and continued presence in the bath solution (n = 2). Micrograph Inset on the right—representative brightfield image of a cytoplasmatic droplet (Carl Zeiss W Plan-Apochromat 40×, NA 1.0 physiological objective).

Cytoplasmic droplets (CDs) were prepared as previously described^1^. Sporangiophores, left in continuity with mycelium in glass vial, were submerged in microscopic chamber with extracellular solution and rapidly cut in the growth zone, 2 mm under the sporangium, to release the content. Most droplets routinely formed within a first minute after the cutting. CDs can be clearly visualized as round, membrane enclosed structures with granular cytoplasm, in various sizes (see inset on the right in Fig. 8). Obtained CDs with clean membranes and diameters between 25 and 45 µm were selected for patch clamp pipette approach. In order to increase the success rate of stable pipette-membrane contacts, droplets were immobilized by coating glass bottom of the microscope chamber with concanavalin-A (100 µg/ml). ConA (Sigma Aldrich) showed the highest capacity to immobilize droplets, compared to collagen (type I, Corning) or poly-l lysine and laminin combined (both Sigma Aldrich). Prior to start of patch clamp measurements, we waited 20 min to allow droplets to settle on the coated chamber bottom, and when necessary, chamber was washed with fresh extracelullar solution, to remove cellular debris and spores released from sporangium upon immersion in solution.

### Characterization of cytoplasmic droplets membrane by current clamp

Membrane potential measured in APW was (mean ± SD) − 64 ± 28 mV (n = 10). Membrane potential measured in standard extracellular and pipette solutions for osmotic stimulation of ORIC was -68 ± 10 mV (n = 8). Sensitivity of membrane potential to 100 μM sodium vanadate was tested in current clamp mode of patch clamp recordings from prepared CDs. Upon addition of vanadate, observed membrane potential change was always in depolarizing direction, as shown in Fig. 8 (left): ΔV = 43 ± 10 mV (n = 3). The sodium vanadate stock solution used was more alkaline (pH 10) than APW (pH 6). To make sure that the observed effect on the membrane potential was induced by vanadate and not by an increase of pH in non-buffered solution caused by addition of vanadate, we have recorded membrane potential in response to application of KOH, inducing exactly the same pH change as vanadate addition Fig. 8 (middle), n = 2. The pH change induced by KOH and corresponding to pH change induced by application of sodium vanadate, exerts an opposing effect, in hyperpolarizing direction, demonstrating that vanadate-induced depolarization is not related to pH effect.

### Cytoplasmic droplets organelle staining and two-photon imaging

The vital dye Rhodamine 123 (Rh123) (2-(6-amino-3-imino-3H-xanthen-9-yl) benzoic acid methyl ester, Sigma-Aldrich) and DAPI (4′,6-diamidino-2-phenylindole, Sigma-Aldrich), were used to stain/visualize mitochondria and nuclei in CDs. Both staining procedures were performed at room temperature (20 °C), without fixation and washing. For mitochondria staining, Rh123 was used at a final concentration of 5 μM in standard solution and imaged after 30 min of incubation^24^. DAPI was used at a concentration of 0.4 μM and imaged after 10 min. The working stock solutions of both dyes were diluted directly into standard extracellular solution. Prior to imaging, the specimen (round cover slip with Concanavalin A-immobilized CDs) was placed at the bottom of the recording chamber or between coverslip (#1.5) and deck glass (Menzel Gläser, Germany) separated with a 300 µm thick glass spacer in order to avoid damaging of the specimen.

The images of cell organelles in CDs were obtained using a homemade nonlinear laser-scanning microscope, as previously described^67,68^. A tunable (700–900 nm) mode-locked Ti:Sapphire femtosecond laser (Mira 900-F, Coherent Inc. CA, USA, pulse duration 160 fs and repetition rate 76 MHz) was used to generate two-photon excitation fluorescence (TPEF) images. The beam was tightly focused in the sample by a high numerical aperture (NA) objective lens (Carl Zeiss, EC Plan-Neofluar 40×, NA 1.3 oil immersion or Carl Zeiss W Plan-Apochromat 40×, NA 1.0 physiological objective). For two-photon excitation and detection of NAD(P)H autofluorescent signal, we used the protocol we developed for *Phycomyces blakesleeanus* hypha NAD(P)H imaging^69^, using excitation and detection conditions (two-photon excitation at 730 nm, visible bandpass filter (400–700 nm) combined with 479/40 bandpass interference filter), as established in literature^70^. For two-photon excitation of Rh123 dye the wavelength of 800 nm was used and the signal was detected through the bandpass interference filter MF530/43 (ThorLabs, USA). The TPEF signal from DAPI was detected through a bandpass interference filter MF479/40 (ThorLabs, USA) and it was excited at 730 nm, as one of the TPEF excitation maxima for DAPI according to literature^71^. A VIS (Visible Bandpass) (400–700 nm) band pass filter, positioned in front of the detector (photomultiplier tube), was used to remove scattered laser light. The average laser power on the sample/specimens during experiments was 4 mW for NAD(P)H autoTPEF, 3 mW for Rh123 and 5 mW for DAPI.

### Patch clamp recordings of ORIC

Pipettes were pulled from thick-walled borosilicate glass with filament (GB150F 0.86 × 1.50 × 100 mm, Science Products and Sutter) on P97 automatic horizontal pipette puller (Sutter Instruments). Pipettes had 5–7 MΩ resistance, to allow seal formation and breaking of the membrane using light suction without degrading the droplets. Pipettes were fire-polished using microforge system (L/MCPZ 101, *List Medical-Elektronic*). Microscope chamber containing prepared CDs was mounted on inverted microscope (Zeiss Axiovert 10, Germany) with manual Luis&Newman micromanipulator. Currents were measured by AM Systems 2400 amplifier (*AM Systems,* USA), and Axopatch 200B (Molecular Devices*,* USA), digitized by Digidata 1200/1550 (Molecular Devices, USA) at 10 kHz, low-pass filtered at 3 kHz and recorded in Clampex 10/11.2 software (Molecular Devices USA).

### Recording protocols

Upon entering the whole cell configuration, the standard voltage-clamp protocol to evoke ORIC was applied: holding voltage − 50 mV, followed by a series of steps − 110 mV to 90 mV, in 20 mV increments. The duration of each step was 500 ms, with a rest period of 0.5–1 s. To record the voltage and time-dependent recovery of ORIC from depolarization-induced inactivation, sweeps of two consecutive depolarizing steps at 70 mV, with varied duration (10–700 ms) and voltage level (− 130, − 50 and 0 mV) in-between the steps were applied (P1/P2 protocol). Series resistance was not compensated. Membrane and access resistance were routinely monitored between recordings. Exclusion criteria for whole-cell configuration recordings from CD were: Rm/Ra < 5, Ra varied more that 20% throughout the experiment. Rm values routinely increased with the blockage of ORIC, as expected based on the previous data^1^. Membrane potential was recorded in current clamp mode, flanked with voltage-clamp recordings used for ensuring seal quality as described above.

### Data analysis

Clampfit 10/11.2 (Molecular Devices USA) software was used for current measurements and fitting of inactivation speed. For run-down measurements, current amplitudes at + 70 mV at the beginning of the response to step voltage pulse (defined as peak current, I_p_) were normalized to the maximum value and plotted as the function of time. For I_ss_ (steady state current), current amplitude at the end of response to test pulse was measured. For comparisons between groups, we used recordings made in the third minute after break-in to whole cell configuration. That was necessary in order to ensure that ORIC is fully osmotically activated by dialysis with hyperosmotic pipette solution, as determined previously^1^. To examine the properties of recovery from voltage induced inactivation using P1P2 protocol, we calculated the ratio of the I_p_ in response to the two subsequent depolarizing steps as I_p_(P2)/I_p_(P1), and exponential fit was used in Graphpad software. To measure inactivation speed of the current, exponential fit was used in Clampfit software. Inactivation and recovery from inactivation were measured as time constant of inactivation (τ_in_) or recovery from inactivation (τ_r_) and rate constant of inactivation (λ = 1/τ_in_) obtained by fitting one component exponential function:

1 $${Y\left(t\right)= {Y}_{0} e}^{t/\tau }+C,$$

where for inactivation speed measurement, Y(t) is measured current, t is time, τ is time constant τ_in_,Y_0_ is value of current at t = 0 and C is steady state value of current amplitude; For recovery from inactivation measurement, Y = P2/P1 (current amplitude at test pulse P2, divided by current amplitude at first pulse P1), Y_0_ is the initial value of P2/P1 at t = 0, τ is time constant τ_r_, t is time of recovery, between P1 and P2 (t_12_) and C is plateau value of maximal achieved recovery, expressed as fraction of P1 amplitude.

To obtain speed of current inactivation, Eq. (1) was fitted to inactivating portion of current recording. To obtain speed of recovery from inactivation, Eq. (1) was fitted to calculated ratio of amplitudes P2/P1 in function of time allowed for recovery (t_12_).

Blocking effect of flavonoids was measured by comparing I_p_ at fixed time points after extracellular flavonoid addition (3 min) with control Ip recorded on the same CD before treatment. Fraction of inactivated current (FIS), defined as (I_p_ −  I_ss_)/I_p_, where I_ss_ (stationary current) is the current amplitude measured at the end of the response to a step protocol, was averaged for each experimental group for each voltage, plotted in function of voltage and fitted by Boltzmann function in Clampfit software (Eq. 2)

2 $$FIS\left(V\right)=FISmin +\frac{FISmax-FISmin}{1+{e}^{{z}_{D F({V}_{0.5}-V)/RT}}},$$

where FIS(V) is FIS at the given voltage V, FIS_max_ and FIS_min_ are the maximum and minimum FIS, V_0.5_ the half-activation potential, z_D_ is the number of gating charges, R the universal gas constant, F the Faraday constant and T the absolute temperature.

GraphPad Prism 6 (San Diego, USA) was used for graphing of obtained data and statistical comparisons. Where data is presented as box and whisker plots, the boxes enclose the 25th and 75th percentile range with the line representing the median and the whiskers extend to the minimal and maximal value. To test statistical significance, groups of data were compared using one-way ANOVA or two-way ANOVA with multiple comparisons and Holm-Sidac correction or unpaired two tailed t-test with Welch's correction for unequal variances. To test statistical significance of differences between fits or between selected parameters of fits, extra sum-of-squares F-test was used with confidence level p < 0.05. Confidence level for statistical significance was: 0.05 (*), 0.01 (**), 0.005 (***), 0.0001 (****).

## Supplementary Information

Supplementary Figures.

## Data Availability

The data is available upon a reasonable request to the corresponding author.
