# Supplementary material for: ATP modulation of osmotically activated anionic current in the membrane of Phycomyces blakesleeanus sporangiophore
Source: Sci Rep. 2023 Jul 24;13:11897. doi: 10.1038/s41598-023-39021-9 (PMC10366193; doi:10.1038/s41598-023-39021-9)
Supplement: Supplementary file 1 — Supplementary Figures. [file 41598_2023_39021_MOESM1_ESM.docx]

Supplemental Fig.1

ORIC with 2 mM GTP (GTP_pip_) decreases with run-down speed indiscernible from the run-down without ATP (no ATP).


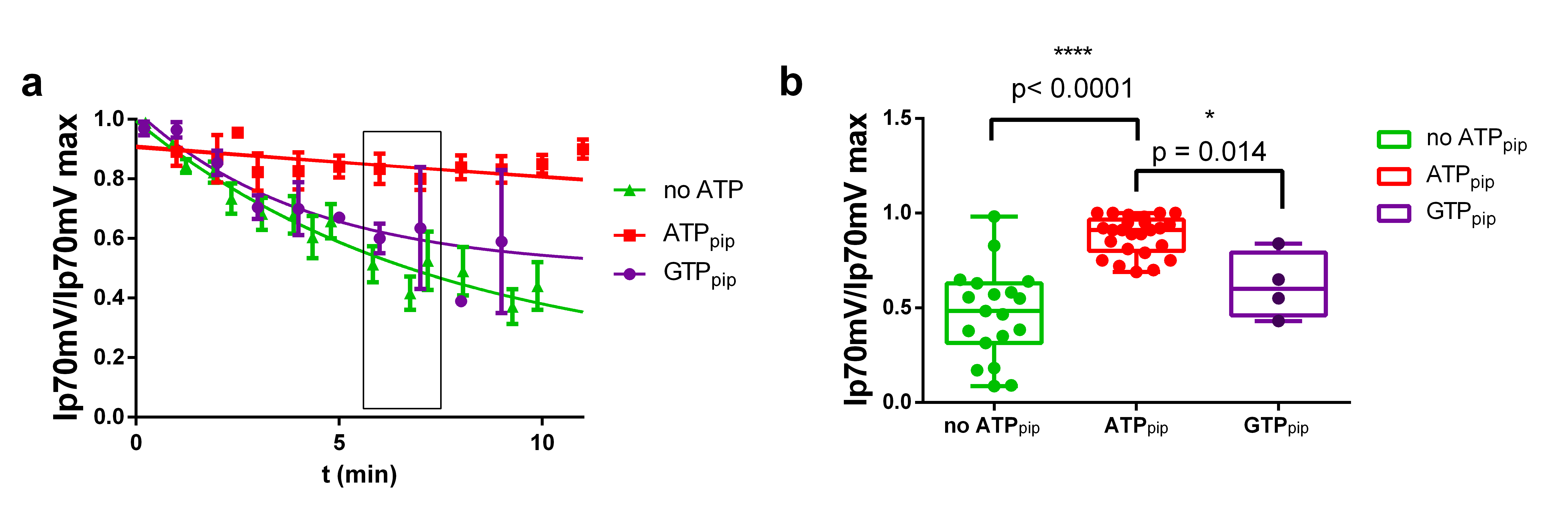
**Suppl. Fig. 1. GTP does not affect ORIC run-down in the same manner as ATP. a.) Time dependence of peak current at +70 mV (Ip 70mV), normalized to maximal value obtained for that individual recording series. Mean ± SE (n =6). Exponential fit of GTP_pip_ series. Extra sum of squares F test of time dependence of GTP and ATP series, p = 0.0182. Groups “no ATP” and “ATP_pip_” are shown for comparison. Rectangle marks the time points used for analysis in graph b. b.) Normalized peak current values at +70 mV, after 6 to 7 min from the start of recording. Box and whisker plots enclosed by the 25th and 75th percentile range, median line with whiskers extending minimal to maximal value, all points shown. ANOVA with Holm Sidac correction**.

Supplemental Fig . 2.


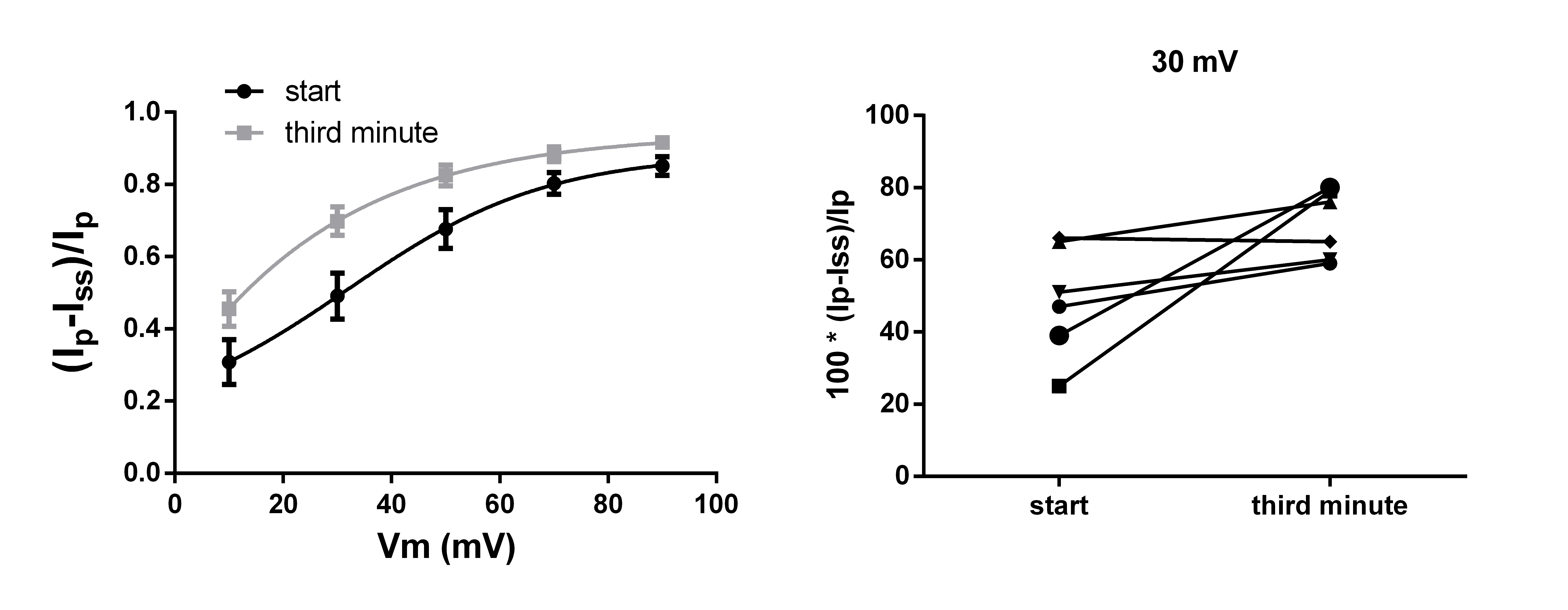
**Suppl. Fig. 2. ORIC inactivation properties change during first three minutes after start of cytoplasmic droplet dialysis with patch pipette content. Left: The** **inactivating current FIC ((Ip-Iss)/Ip) at the beginning of the recording (V_50_ = 19 ± 8 mV), labeled as “start”, and in the third minute of dialysis (V_50_= 5 ± 6 mV), labeled “third minute”. Recorded from the same CDs (n =6). Right: Within-group variability of percent of inactivating current decreases (p = 0.0189) after two whole minutes after whole-cell break-in. Data extracted from the curves in left graph.**
